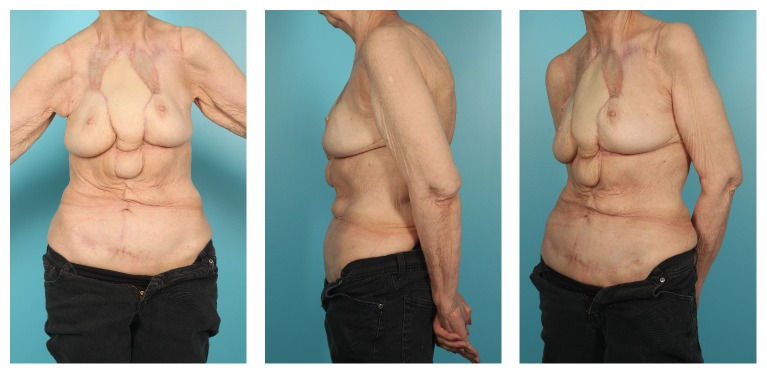# Giant Anterior Chest Wall Basal Cell Carcinoma: An Approach to Palliative Reconstruction

**DOI:** 10.1155/2016/5067817

**Published:** 2016-12-19

**Authors:** Pauline Joy F. Santos, Christina Prendergast, Amber Leis

**Affiliations:** Department of Plastic Surgery, University of California, Irvine, CA, USA

## Abstract

Anterior chest wall giant basal cell carcinoma (GBCC) is a rare skin malignancy that requires a multidisciplinary treatment approach. This case report demonstrates the challenges of anterior chest wall GBCC reconstruction for the purpose of palliative therapy in a 72-year-old female. Surgical resection of the lesion included the manubrium and upper four ribs. The defect was closed with bilateral pectoral advancement flaps, FlexHD, and pedicled VRAM. The palliative nature of this case made hybrid reconstruction more appropriate than rigid sternal reconstruction. In advanced metastatic cancers, the ultimate goals should be to avoid risk for infection and provide adequate coverage for the defect.

## 1. Introduction

Basal cell carcinoma (BCC) is the most common, yet relatively benign and slow-growing, skin malignancy. Contrastingly, giant basal cell carcinoma (GBCC) is a rare and aggressive skin malignancy. GBCC is defined as a tumor with a diameter larger than 5 cm, and it accounts for only 1% of all BCC [1]. While individuals affected by BCC usually have a significant history of sun exposure and are more commonly fair-skinned, male, and older, it is important to recognize that GBCC is more likely to be present for several years, to have previous treatment, or to have radiation exposure. Furthermore, GBCC is characterized by an aggressive histological subtype (morpheaform, micronodular, and metatypical) [2].

In numerous case reports, neglect of the growing GBCC tumor was common and often discovered secondary to another medical problem [3]. In a review of 51 cases of GBCC, peak incidence was found to be in the seventh decade of life. The mean disease duration was 14.5 years, and at the time of presentation, average size was 14.77 cm at the tumor's largest diameter. Additionally, metastasis was reported in 17.6% of the patients at time of presentation and is considered the worst prognostic factor [1]. Despite optimal therapy, defined as wide local excision with histologically confirmed tumor-free margins, recurrence or metastasis developed in 38.3% of the patients. Excision was frequently followed by adjuvant radiochemotherapy, and the overall cure rate was reported to be 61.7% at 2 years [1].

Although GBCCs are rare, anterior chest wall GBCCs are even more uncommon. In the previously mentioned review of 51 cases of GBCC, the majority of cases were located on the head and neck, with only one case on the anterior chest wall area [1]. In a review of 8 cases of GBCC, all tumors were located on the face and scalp, with the exception of one located on the left anterior chest [4].

There are no clear standards for the treatment of GBCC given its rarity. The approach in the 8 case series was a 1-stage aggressive surgical resection with immediate bone and soft tissue reconstruction. Outcomes included free soft tissue margins and relief of pain and hygiene issues associated with the wounds [4]. In another series of cases, patients with GBCC were treated with 3 cycles of metvix photodynamic therapy and a subsequent 6-week course of topical imiquimod to decrease the size of the wound prior to excision [5].

Treatment of GBCC always requires a multidisciplinary approach with the goal of tumor-free margins, which are associated with long-term survival [1, 3]. The suggested adequate margin range is 2.5–3 cm. Of note, chemotherapy or radiotherapy without excision does not achieve local control [1]. Specific treatment for anterior chest wall GBCC is essentially nonexistent, most likely because it is scarcely seen. While most cases are treated with wide local excision and reconstruction with grafting or flaps, the utility of anterior chest wall reconstruction in the context of palliative goals has not been well described.

## 2. Case Description

**Figure 1:**
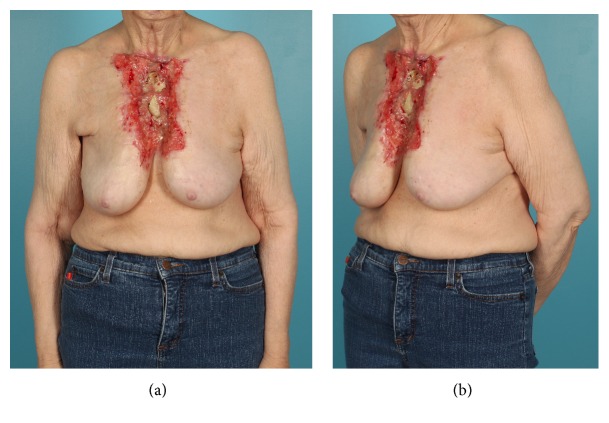


We present the case of a 72-year-old female with a history of hypothyroidism who presented to an outside hospital for transfusion after routine thyroid bloodwork revealed significant anemia. A large ulcerating chest wound was discovered during her evaluation. The patient had not informed any care provider about this wound previously. She was referred to a plastic surgeon for management of her chest wound. Examination at that time revealed a large ulcerating midline chest wound with exposed and denuded sternum and ribs (Figure 1(a)). There were heaped-up erythematous margins at the skin edges, and her chest wound was weeping seropurulent fluid from exposed intercostal spaces (Figure 1(b)). Her breasts appeared contracted toward the midline. There was a palpable right breast mass and also right axillary lymphadenopathy. The patient was unable to provide a concrete assessment as to when her lesion first appeared. However, she believed the wound began after a curling iron burn. The patient denied constitutional symptoms.

## 3. Investigations

A CT of the chest, abdomen, and pelvis identified multiple suspicious pulmonary nodules, an ulcerating soft tissue defect anterior to the sternum, a pathological fracture of the body of the sternum, and right axillary adenopathy. Biopsies were taken of the largest right axillary node and right breast mass. The right axillary node was positive for squamous cell carcinoma. The biopsy from the breast lesion was inconclusive. Biopsies of the chest wound were positive for basal cell carcinoma. FNA of the lung mass was also positive for malignant cells, consistent with squamous cell cancer.

## 4. Treatment

**Figure 2:**
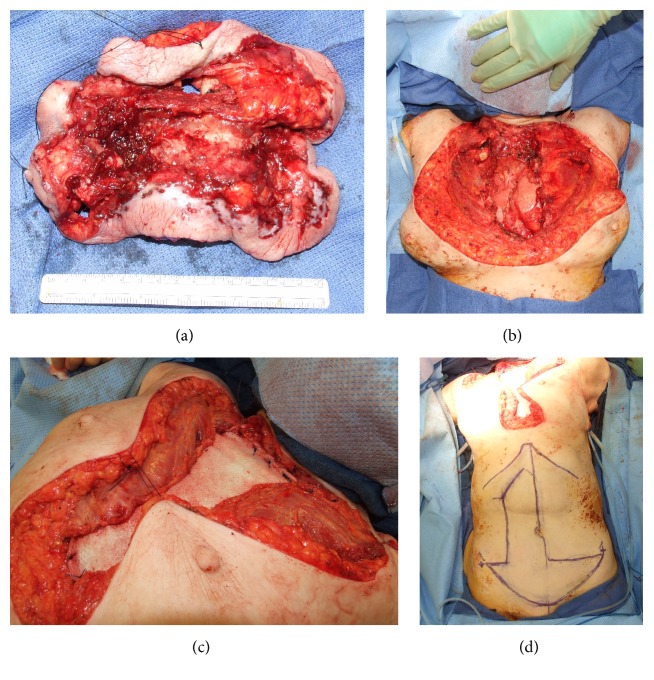


The patient was presented at a multidisciplinary tumor board, and her planned treatment was to be wide local excision with a minimum of 1 cm margin, followed by radiation and chemotherapy. Her treatment was designed to provide palliation, a closed hygienic wound, and to offer improved quality of life. The surgical resection was performed by the Surgical Oncology and Cardiothoracic Surgery services. The right internal mammary artery was preserved for planned reconstruction. Frozen sections were sent to ensure tumor-free margins. The specimen included the manubrium and upper four ribs (Figure 2(a)), leaving a bony defect 8 × 13 cm in the chest wall (Figure 2(b)). The inferior ribs and xiphoid were left intact. The soft tissue portion of the specimen measured 17 × 15 cm, while the soft tissue defect of the chest measured 30 cm in width and 25 cm in length, extending up onto the neck and out on to the clavicles, likely due to contracted nature of the wound. The tumor and specimen involved the medial aspect of the bilateral pectoralis major muscles.

The sternal defect was closed with bilateral pectoral island advancement flaps, which did not reach to the midline secondary to the partial resection of the muscle (Figure 2(c)). The muscle was secured to the ribs at the periphery of the bony defect. Sternal reconstruction was supplemented by 1.6 mm thick structural FlexHD, also secured to the ribs. A pedicled vertical rectus abdominis muscle (VRAM) from the right abdomen was designed with a transverse extension to allow for maximal wound coverage (Figure 2(d)). Intraoperative SPY angiography confirmed the viability of this design. The DIEA and VC were dissected and preserved for supercharging. After transposition and inset, the venous anastomoses were performed bilaterally into the available neck vessels.

## 5. Outcome and Follow-Up

The patient was admitted to the intensive care unit for routine monitoring in the perioperative period. The patient met all the postoperative milestones and was discharged to a skilled nursing facility on postoperative day 11. She continued to improve and was followed weekly in clinic. At her third postoperative visit, on postoperative day 29, she was noted to have a superior dehiscence with purulent fluid draining from underneath the flap. The patient did not appear to recognize that this was abnormal. She was admitted to the hospital and underwent several operative washout and debridement procedures and was successfully reclosed after 2 weeks. Biopsies taken at suspicious appearing skin margins were free of tumor. Patient is now on oral chemotherapy, and her postoperative pictures demonstrate successful palliative coverage after resection of her anterior chest wall GBCC (Figure 3).

## 6. Discussion

Our patient, like most case reports of GBCC, did not seek treatment for her growing lesion. Unlike other case reports of anterior chest wall GBCC, our patient presented with a history of a burn. Of the 51 case series of GBCCs, only one patient had a history of a burn [1]. Our patient's burn was suspicious for Marjolin's ulcer given her squamous cell metastasis to the lungs. Of note, marjolin's ulcers are epidermoid carcinomas that develop on nonhealing scar tissue. In a review of 51 patients with Marjolin ulcers, 43 patients presented with squamous cell carcinoma, and only one patient presented with basal cell carcinoma. The one patient presenting with basal cell carcinoma did not exhibit metastasis [6]. Our patient has an interesting presentation in that her chest wound exhibited basal cell carcinoma, and she had squamous cell metastasis.

A multidisciplinary approach is critical for successful resection and reconstruction [1]. For our patient, it was imperative to coordinate with the Surgical Oncology and Cardiothoracic Surgery services given her advanced cancer presentation and the palliative nature of the procedure. This strategy ensured proper management of surgical resection, evaluation of cancer metastasis, and preservation of tissue and anatomy for successful reconstruction.

Treatment of chest wall resection and reconstruction relies on a comprehensive understanding of reconstructive techniques and a variety of secondary options if the original plan is not adequate. Techniques include the use of muscle and musculocutaneous flaps, free microvascular transfers, and prosthetic material [7]. A combination of techniques is often used, including our patient's treatment. Our patient's case demonstrates the importance of anticipating larger than expected defects and the need for adequate options for reconstruction.

One can appreciate the variety of approaches for the treatment of anterior chest wall GBCC when reviewing the literature. Each case report describes a different combination of approches and techniques. One case describes a patient with an anterior chest wall GBCC that metastasized from the abdominal wall. The defect was closed with a left latissimus dorsi free flap, with anastomosis to the inferior thyroid artery, as well as a split thickness skin grafting [8]. Another case described a 13.5 cm diameter lesion on the left lower anterior thoracic wall. The defect on the chest was reconstructed with prolene mesh, porcine acellular dermis, an omental flap, and pedicled vertical rectus abdominus myocutaneous flap [9]. One case utilized an approach that encompassed components of the prior cases described. The surgeons used a 2-mm Gore-Tex patch and full-thickness rotation left latissimus dorsi flap with additional split skin grafting [10]. In a case where the central anterior chest wall defect measured 10 × 6 cm, repair included an omental flap, Simplex bone cement, Gore-Tex mesh, and Marlex mesh. A split thickness skin graft was placed, and a vacuum-assisted closure device covered the wound [11]. Unfortunately, in one case, surgical excision was not performed because of left brachiocephalic vein occlusion [12]. Reconstruction is highly dependent on the extent of the defect.

In our case, we chose FlexHD because of her high risk of infection and no indication for traditional rigid sternal reconstruction with materials like Marlex methyl methacrylate or titanium. Of note, the use of acellular dermal matrix mesh in the setting of contamination in hernia repair is well-established [13]. Therefore, it was appropriate for use in this case of high risk of infection. Additionally, although traditional rigid sternal reconstructions may reduce hospitalization days and time on the ventilator, patients with extensive chest wall resections, such as in our case, do not have a compromise of pulmonary function [7]. Furthermore, the use of synthetic mesh has been shown to improve chest wall stability and reduce ventilator dependence [14].

## 7. Complications

At the time of return to OR, we found the FlexHD incorporated into the rectus muscle, but not the underlying sternal defect. Significant fibrosis was noted between the rib margins, with no chest wall instability. Traditional teaching for rigid sternal reconstructions may not need to be followed in cases such as ours, with palliation as the goal and high risk for infection.

## 8. Conclusions

Anterior chest wall GBCC is infrequent amongst GBCC and results in large defects that are challenging to repair. Our patient's case was complicated by squamous cell metastasis to the lung, which is a unique presentation compared to other cases of anterior chest wall GBCC requiring a multidisciplinary approach. For our patient and most other cases of GBCC, wide excision with immediate reconstruction provides an increase in quality of life. Our patient is doing well postoperatively; however, close follow-up is required in these cases. Her neglect of her original lesion is likely to reflect in her postoperative self-care.

## Figures and Tables

**Figure 3:**